# The BRAF^V600E^ mutation analysis and risk stratification in papillary thyroid carcinoma

**DOI:** 10.20945/2359-3997000000285

**Published:** 2020-08-24

**Authors:** Rafael Selbach Scheffel, Ana Patrícia de Cristo, Mirian Romitti, Carla Vaz Ferreira Vargas, Lucieli Ceolin, André B. Zanella, Jose Miguel Dora, Ana Luiza Maia

**Affiliations:** 1 Universidade Federal do Rio Grande do Sul Faculdade de Medicina Hospital de Clínicas de Porto Alegre Porto Alegre RS Brasil Unidade de Tireoide, Hospital de Clínicas de Porto Alegre, Faculdade de Medicina, Universidade Federal do Rio Grande do Sul, Porto Alegre, RS, Brasil; 2 Universidade Federal do Rio Grande do Sul Instituto de Ciências Básicas da Saúde Departamento de Farmacologia Porto Alegre RS Brasil Departamento de Farmacologia, Instituto de Ciências Básicas da Saúde, Universidade Federal do Rio Grande do Sul, Porto Alegre, RS, Brasil; 3 Université Libre de Bruxelles Institut de Recherche Interdisciplinaire en Biologie Humaine et Moléculaire Brussels Belgium Institut de Recherche Interdisciplinaire en Biologie Humaine et Moléculaire, Université Libre de Bruxelles, Brussels, Belgium

**Keywords:** Papillary thyroid carcinoma, BRAF mutation, risk classification, persistent disease

## Abstract

**Objective:**

Although the prognostic role of BRAF^V600E^ mutation in papillary thyroid carcinoma (PTC) is controversial, the American Thyroid Association (ATA) includes the mutational status in their risk stratification system. To evaluate the impact of the BRAF^V600E^ mutation status on PTC risk stratification.

**Subjects and methods:**

PTC patients attending a university-based hospital who had the analysis of the BRAF^V600E^ mutation were included. Persistent disease was defined as the presence of biochemical or structural disease. The performance of the ATA risk stratification system on predicting persistent disease with or without the BRAF^V600E^ mutation status information was evaluated.

**Results:**

Of the 134 patients evaluated, 44 (32.8%) carried BRAF^V600E^ mutation. The median tumor size was 1.7 cm (P25-75 1.0-3.0), 64 (47.8%) patients had lymph node, and 11 (8.2%) distant metastases. According to the ATA risk stratification system, patients were classified as low, intermediate, and high risk in 55 (41%), 59 (44%), and 20 (14%) patients, respectively. The data on BRAF^V600E^ mutation reclassified 12 (8.9%) patients from low to intermediate risk. After a median follow-up of 8.5 years, the prevalence of persistent disease was similar in patients with and without BRAF^V600E^ mutation (P = 0.42). Multivariate analysis failed to demonstrate an association between the BRAF^V600E^ mutation and persistent disease status (RR 0.96; 95%CI 0.47-1.94). Notably, none of the patients reclassified from low to intermediate risk showed persistent disease on follow-up.

**Conclusion:**

Inclusion of BRAF^V600E^ mutational status has a limited impact on risk stratification and does not add to the prediction of outcomes in PTC patients.

## INTRODUCTION

Differentiated thyroid cancer (DTC) is the most common malignancy in endocrine organs, and its incidence is increasing worldwide ( 1 ). The appropriate management of DTC patients, especially those with papillary thyroid carcinoma (PTC), has been changing in recent years and is still a matter of debate. Accurate prognostic factors, which could lead to a more individualized approach, are required and molecular markers have been proposed as potential key elements on the decision-making process ( 2 ).

*BRAF* is one of the most studied genes in PTC pathogenesis. *BRAF* mutations activate the mitogen-activated protein kinase (MAPK) pathway, resulting in increases in cell proliferation, dedifferentiation, and apoptosis ( 3 ). The BRAF point mutation T1799A is the most common, resulting in the exchange of valine (V) to glutamic acid (E) at residue 600 (BRAF^V600E^). The first studies describing the role of BRAF^V600E^ mutation on the pathogenesis of PTC were published in 2003 ( 4 ).

From the clinical perspective view, the prognostic role of BRAF^V600E^ mutation is still controversial ( 5 ). Several studies were focused on searching for an association between BRAF^V600E^ mutation and features related to unfavorable course of the disease and showed conflicting results ( 6 – 8 ). Several studies have shown an association of mutated BRAF^V600E^ with lymph node metastases, extrathyroidal extension, tumor size, and advanced disease stage ( 9 ). However, the impact of the BRAF^V600E^ mutation on the clinical outcomes (persistent disease, recurrence, survival) is not so clear. In a pivotal multicenter cohort, Xing and cols. showed an association of mutated BRAF^V600E^ with increased PTC disease-specific mortality on univariate analysis but failed to demonstrate independent association in a multivariable model adjusting for patient sex, age at diagnosis, medical center, and several conventional pathologic factors ( 10 ). A second study, with a similar design but including a larger number of patients (n = 2099), showed an independent association between BRAF^V600E^ mutation and recurrent disease both in the overall PTC population and after stratification for histological subtypes (classic and follicular variant) ( 11 ). Similar results were reached in a recent systematic review ( 7 ). Nevertheless, as stated by the authors, the findings should be interpreted with caution due to heterogeneity of the data, which include differences in the patient demographics and ethnicities, as well as in the therapeutic approach and follow-up ( 7 ).

Despite these limitations, the 2015 American Thyroid Association (ATA) guidelines had included the BRAF^V600E^ mutation status in the stratification system for persistent/recurrent disease according to the ‘continuum of risk’ model. In this proposal, those patients classified as low risk-disease with intrathyroidal PTC lesions of 1-4 cm should be reclassified as intermediate risk if positive BRAF^V600E^ mutation is known ( 12 ). Here, we aimed to evaluate the impact of the BRAF^V600E^ mutation status on risk stratification as well as on disease outcomes in a cohort of patients with PTC in a tertiary, university-based referral center.

## SUBJECTS AND METHODS

### Patients and study design

The patients included were followed in a cohort of DTC patients from the Thyroid Outpatient Clinic of the Thyroid Unit, of Hospital de Clínicas de Porto Alegre (HCPA), a tertiary care, university teaching hospital in southern Brazil. From 2009 to 2015, all consecutive patients with a histological diagnosis of PTC who had paraffin-embedded tumor tissue available for analysis of the BRAF^V600E^ mutation were included in this study.

### Treatment protocol and follow-up

Our DTC treatment protocol consists of performing total thyroidectomy (TT), followed or not by administration of radioactive iodine (RAI) as indicated, and the use of suppressive levothyroxine therapy according to current guidelines ( 12 ). The same surgical team operated all patients and decisions regarding cervical lymph node dissection were taken at the discretion of the surgical team. The iodine administration protocol used RAI activities prescribed at the attending physician's discretion. RAI was administered in a stimulated thyrotropin (TSH) condition of endogenous hypothyroidism (TSH > 30 mUI/L), after withdrawing levothyroxine (at least 3-4 weeks) ( 13 ). A post-therapy whole-body scan (WBS) was performed seven to ten days after the RAI administration. Of note, the information on BRAF^V600E^ mutation status was not used to decide RAI indication in these patients.

In the first evaluation, the following data were recorded for each patient: demographics, tumor characteristics (e.g., histological features, extension, and lymph node involvement) and treatment (e.g., surgery, RAI, and other interventions). Each patient was classified using the 8th edition of the TNM/AJCC (TNM8) staging system (I, II, III, or IV) ( 14 , 15 ). N0 status was determined by clinical examination of the neck or preoperative and postoperative neck ultrasound (US) imaging or macroscopic examination during surgery and pathological examination of patients with lymph node resection.

The follow-up protocol called for an initial assessment at 3 to 6 months post-surgery, which included physical examination of the neck and measurements of serum thyroglobulin (Tg) levels under TSH suppression (Tg-T4) and antithyroglobulin antibody (TgAb). In a second evaluation, 6 to 12 months after the initial treatment, serum Tg was measured under conditions of a stimulated TSH (sTg) with endogenous hypothyroidism (TSH > 30 mIU/L) as indicated by Tg-T4 levels. Neck ultrasound (US) was also performed during the first year of follow-up. Patients classified as excellent response (see below) were scheduled for annual visits that included physical examination of the neck and measurements of Tg-T4 and TgAb. Patients with indeterminate or persistent disease were scheduled for medical visits twice a year and evaluated for additional therapy as needed, and based on disease extension, cumulative RAI activity, and previous response to therapy. Additional imaging studies [e.g., neck US, diagnostic I-131 whole-body scan (WBS) and computed tomography (CT)] as indicated when clinical or laboratory findings raised suspicion of persistent or recurrent disease. The duration of follow-up was defined as the time between the TT and the last medical visit to the clinic.

### Mutation analysis

BRAF^V600E^ mutation analysis was performed in genomic DNA extracted from 10-µm slides of paraffin-embedded tissue blocks using the Magnesil Genomic Fixed Tissue System (Promega Corporation, Madison, USA) according to the manufacturer's instructions. BRAF exon 15 was amplified by PCR using specific oligonucleotides: 5’-ACCTAAACTCTTCATAATGCTTGCT-3’ and 3’- CTGATTTTTGTGAATACTGGGAACT-5’. To PCR amplification 100–300 ng/µL of DNA were used in a reaction mix (25 µL) containing 20 mM Tris-HCl, 50 mM KCl, 2 mM MgCl2, 0.2 mM dNTPs, 0.2mM of each primer, and 1.25 U Platinum Taq DNA Polymerase (Invitrogen, Thermo Scientific, São Paulo, Brazil). For sequencing, PCR products were purified using the GFX PCR DNA purification kit (GE Healthcare, Buckinghamshire, UK) and submitted to direct sequencing using the Big Dye Terminator Cycle Sequencing Ready Reaction Kit (Applied Biosystems, Foster City, CA, USA).

### Outcomes

Disease status was defined based on clinical examination, Tg-T4 and/or sTg levels, neck US, post-RAI WBS (when available), and additional imaging exams when indicated. Patients were classified into four categories: excellent response, indeterminate response, biochemical incomplete response, or structural incomplete response.

In patients submitted to RAI, excellent response was defined as negative imaging and Tg-T4 < 0.2 ng/mL or sTg < 1.0 ng/mL. Biochemical incomplete response was defined as negative imaging and Tg-T4 > 1.0 ng/mL or sTg > 10.0 ng/mL or rising TgAb levels. Structural incomplete response was defined as structural or functional evidence of disease with any Tg or TgAb level. Indeterminate response was defined as non-specific findings on imaging studies or Tg-T4 between 0.2 to 1.0 ng/mL or sTg between 1.0 and 10.0 ng/mL or TgAb stable or declining.

In patients not submitted to RAI, excellent response was defined as negative imaging and Tg-T4 < 0.2 ng/mL or sTg < 2.0 ng/mL. Biochemical incomplete response was defined as negative imaging and Tg-T4 > 5.0 ng/mL or sTg > 10.0 ng/mL or increasing Tg levels over time or rising TgAb levels. Structural incomplete response was defined as structural or functional evidence of disease with any Tg or TgAb level. Indeterminate response was defined as non-specific findings on imaging studies or Tg-T4 between 0.2 to 5.0 ng/mL or sTg between 2.0 and 10.0 ng/mL or TgAb stable or declining.

### Laboratory analysis

Serum Tg measurements were conducted using immunoradiometric assays (from 2000 to 2002 radioimmunoassay; 2002 to 2010 electrochemiluminescence and 2010 until the present chemiluminescence) that indicated a functional sensitivity of 0.2 ng/mL. TgAb were measured using the passive agglutination method from 2000 to 2010 and by chemiluminescence from 2010 until the present. TSH levels were measured by chemiluminescence assay from 2000 to 2006 (Immulite 2000 SIEMENS, Munich, Germany), electrochemiluminescence from 2006 to 2010 (Modular E ROCHE, Basel, Switzerland), chemiluminescence assay from 2010 to 2014 (Centaur XP SIEMENS, Munich, Germany) and electrochemiluminescence from 2014 until the present (Cobas E602 ROCHE, Basel, Switzerland). After each new assay had been implemented, the necessary procedures for standardization and validation were performed. These tests were all conducted in the HCPA central laboratory.

### Statistical analysis

The clinical and laboratory data are reported as the mean ± standard deviation (SD) values or as the median and percentiles 25 and 75 (P25-75) for continuous variables or as absolute numbers and percentages for categorical variables. Comparative analyses of frequencies were performed using Pearson Chi-Square or Fisher's Exact Test, as appropriate. These analyses were performed using the Statistical Package for Social Science Professional software version 20.0 (IBM Corp., Armonk, NY, USA). All tests were two-tailed, and a P < 0.05 was considered statistically significant.

Univariate analysis and generalized linear models with a log link and Poisson errors were used to estimate the impact of clinical variables and B-RAF^V600E^ mutation status as potential prognostic factors for persistent disease.

## RESULTS

### Patients

From a cohort of 681 PTC patients ( 16 ), 134 individuals who had paraffin-embedded tumor tissue and BRAF^V600E^ mutation analysis were included. Clinical and oncological features of the study subjects are summarized in Table 1 . The mean age at the time of diagnosis was 45.2 ± 15.8 years, and 105 (78.4%) were women. Median tumor size was 1.7 cm (P25-75 1.0-3.0); 64 (47.8%) patients had lymph node metastases, and 11 (8.2%) patients had distant metastases. The TNM/AJCC classification was as follows: 111 (82.8%) patients were stage I, 17 (12.7%) were stage II, 2 (1.5%) were stage III, and 4 (3.0%) stage IV. The clinicopathological features (sex, age, TNM stage, and ATA risk stratification) of this subgroup of 134 patients were similar to the whole cohort (all P > 0.05).

**Table 1:** Characteristics of 134 patients with papillary thyroid carcinoma and BRAF mutation analysis

Age at time of diagnosis (years)		45.2 ± 15.8
Female sex – n (%)		105 (78.4)
Tumor size (cm)		1.7 (1.0-3.0)
Gross extrathyroidal extension – n (%)		16 (11.9)
Lymph node metastasis – n (%)		
	N0	67 (50.0)
	N1a	21 (15.7)
	N1b	43 (32.1)
	Nx	3 (2.2)
Distant metastasis – n (%)		11 (8.2)
TNM AJCC stage – n (%)		
	I	111 (82.8)
	II	17 (12.7)
	III	2 (1.5)
	IV	4 (3.0)
2009 ATA risk level – n (%)		
	Low	55 (41)
	Intermediate	59 (44)
	High	20 (14)
Positive BRAF mutation – n (%)		44 (32.8)
RAI therapy – n (%)		115 (85.8)
Follow-up (years)		8.5 (5.0-12.0)

Data are expressed as the mean ± SD, median (percentiles 25-75), or frequencies.

N0: No evidence of lymph node metastasis; N1a: metastasis to level VI or VII lymph nodes; N1b: metastasis to lateral neck lymph nodes; Nx: regional lymph nodes not accessed.

TNM/AJCC: TNM staging system of the American Joint Committee on Cancer.

ATA: American Thyroid Association; RAI: radioactive iodine.

One-hundred-fifteen patients (85.8%) received ablative or therapeutic RAI. Post-therapy WBS was performed in 110 patients: 4 (3.6%) showed no uptake, 98 (89.2%) presented only cervical uptake, and 8 (7.2%) distant uptake.

### Impact of BRAF mutation status on clinicopathological features and ATA risk stratification system

The prevalence of BRAF^V600E^ mutation was 32.8% (44/134 patients, 95% CI 25-42). To investigate whether the presence of BRAF^V600E^ mutation would impact on clinicopathological features, the patients were grouped according to their BRAF^V600E^ mutation status. Positive BRAF^V600E^ patients had larger tumors (median size 2.2 vs. 1.6 cm; P = 0.03) and a trend towards more gross extrathyroidal extension of the tumor (20.5 vs. 7.8%; P = 0.06). However, no differences were observed between the groups for sex, age, cervical or distant metastasis, TNM AJCC stage, or ATA risk classification ( Table 2 ).

**Table 2:** Clinical and oncological characteristics of patients with positive or negative BRAF mutation

		Positive BRAF mutation (n = 44)	Negative BRAF mutation (n = 90)	P
Age at time of diagnosis (years)		45.7 ± 13.8	44.9 ± 16.7	0.79
Female sex – n (%)		36 (81.8)	69 (76.6)	0.65
Tumor size (cm)		2.2 (1.5 – 3.8)	1.6 (0.9 – 2.6)	0.03
Gross extrathyroidal extension – n (%)		9 (20.5)	7 (7.8)	0.06
Lymph node metastasis – n (%)				0.32
	N0	18 (40.9)	49 (54.5)	
	N1a	8 (18.2)	13 (14.4)	
	N1b	18 (40.9)	25 (27.8)	
	Nx	0	3 (3.3)	
Distant metastasis – n (%)		3 (6.8)	8 (8.9)	0.68
TNM AJCC stage – n (%)				0.23
	I	36 (81.8)	75 (83.8)	
	II	5 (11.4)	12 (13.3)	
	III	0	2 (2.2)	
	IV	3 (6.8)	1 (1.1)	
2009 ATA risk – n (%)				0.34
	Low	15 (34.1)	40 (44.4)	
	Intermediate	20 (45.5)	39 (43.3)	
	High	9 (20.5)	11 (12.2)	
RAI therapy – n (%)		36 (81.8)	79 (88.8)	0.49

Data is expressed as mean ± SD, median (percentiles 25-75) or frequencies.

N0: No evidence of lymph node metastasis; N1a: metastasis to level VI or VII lymph nodes; N1b: metastasis to lateral neck lymph nodes; Nx: regional lymph nodes not accessed.

TNM/AJCC: TNM staging system of the American Joint Committee on Cancer.

ATA: American Thyroid Association.

RAI: Radioactive iodine.

According to the 2009 ATA risk stratification system, the risk level was low in 55 (41%) patients, intermediate in 59 (44%) patients, and high in 20 (14%) patients. In the group of intermediate risk, the more common determinant to classify patients in this risk category was the presence of lymph nodes metastasis (n = 48, 81.3%), followed by microscopic extrathyroidal invasion (n = 23, 38.9%). In the high-risk group, the main determinant was the presence of distant metastasis (n = 11; 55%), followed by macroscopic invasion (n = 9, 45%). When we included the BRAF^V600E^ mutation status to the ATA risk stratification system, we reclassified 12 (8.9%) patients from low to intermediate risk. All cases consisted of intrathyroidal PTC tumors (size < 4 cm, restricted to the thyroid) ( Table 3 ).

**Table 3:** Clinical and oncological characteristics of the 12 (8.9%) patients reclassified from low to intermediate risk

Sex, age at diagnosis	Tumor size (cm)	Lymph node metastasis	TNM stage	Follow-up status and duration (years)
Female, 48	1.0	0a	I	Indeterminate (12)
Female, 53	1.5	0a	I	Excellent (4)
Female, 51	5.5	0b	I	Indeterminate (9)
Female, 71	1.1	0a	I	Excellent (8)
Female, 41	2.7	0a	I	NA
Male, 25	2.5	0a	I	Excellent (6)
Female, 60	2.0	0a	I	Indeterminate (6)
Female, 61	1.5	0b	I	Excellent (7)
Female, 39	2.0	0b	I	Excellent (5)
Female, 24	3.9	0b	I	Indeterminate (5)
Male, 79	2.1	0a	I	Indeterminate (4)
Female, 49	1.6	0a	I	Indeterminate (5)

TNM/AJCC: TNM staging system of the American Joint Committee on Cancer.

0a: one or more cytological or histologically confirmed benign lymph nodes; 0b: no radiological or clinical evidence of lymph nodes metastasis.

NA: Not available.

### Impact of BRAF^V600E^ mutation status on clinical outcomes

After a median follow-up of 8.5 years (P25-75 5.0-12.0), disease status was available for 125 patients: 56 (60.8%) had excellent response, 27 (21.6%) indeterminate response, 3 (2.4%) incomplete response, and 19 (15.2%) structural incomplete response.

The prevalence of persistent disease (biochemical and structural) was not different when comparing patients with positive BRAF^V600E^ mutation and those without the mutation: 23.8 vs. 14.5%, P = 0.42. Notably, none of the patients who had been reclassified from low to intermediate risk due to their mutational status information showed persistent disease on follow-up ( Table 3 ). We were unable to evaluate the impact of BRAF^V600E^ mutation on RAI response, due the low number of positive BRAF^V600E^ patients who did not receive RAI dosing (n = 7).

Additional analyses using a multivariate model including disease status as the dependent variable and sex, tumor size, lymph node and distant metastasis, and BRAF^V600E^ mutation as independent variables are shown in Table 4 . Tumor size, lymph node and distant metastasis were independent risk factors for persistent disease. Positive BRAF^V600E^ mutation was not associated with persistent disease (RR 0.96; 95% CI 0.47-1.94).

**Table 4:** Multivariate analysis of predictors of persistent disease status

	RR (95% CI)	P
Male sex	1.44 (0.78-2.63)	0.23
Tumor size	1.19 (1.03-1.39)	0.01
Lymph node metastasis	2.62 (1.06-6.45)	0.03
Distant metastasis	3.61 (1.89-6.90)	< 0.01
Positive BRAF mutation	0.96 (0.47-1.94)	0.91

## DISCUSSION

The use of specific molecular classifiers as prognostic markers is a research trend in thyroid cancer, and a potential role of BRAF^V600E^ mutation has been long advocated. While the majority of studies demonstrated a relationship between positive BRAF^V600E^ mutation status and poorer clinicopathological features (tumor size, extrathyroidal extension and lymph nodal metastasis), its role as an independent prognostic marker in PTC patients is still a matter of debate ( 9 ). Here, we showed that including the information about the BRAF^V600E^ status to the ATA risk stratification system resulted in increasing the risk for some patients (8.9% of our sample population), although without any impact on disease outcomes. Of note, none of the patients who were reclassified from low to intermediate risk based on BRAF^V600E^ status showed persistent disease on follow-up.

The inclusion of the BRAF^V600E^ mutation analysis in the risk classification system, as suggested by ATA [12], increases the complexity of care and costs without a clear benefit. BRAF^V600E^ mutation does not seem to be an independent prognostic marker and should be analyzed in association with other prognostic factors. Even in case of a real association of BRAF^V600E^ mutation with disease recurrence, the clinical application of BRAF^V600E^ as a prognostic marker is hampered by its low specificity. Indeed, when we look to the performance of the mutation analysis as a diagnostic test for the prediction of recurrent/persistent disease, it has an acceptable sensitivity (60-70%), but poor specificity (34-57%) with a positive predictive positive value of only 7-25% ( 7 , 9 ). Our data showed similar results: sensitivity of 45%, a specificity of 69%, and a positive predictive value of 24%.

We observed a prevalence of positive BRAF^V600E^ mutation of 32.8% in our study. These numbers are comparable to those reported in previous studies carried out in the Southeastern region of Brazil (28.1 to 48.3%) ( 17 , 18 ), but lower than results obtained from the central (63.8%) and Northeast (65.1%) regions ( 19 , 20 ). Indeed, a systematic review of 63 studies (including 20,764 patients) has shown that the prevalence of BRAF^V600E^ mutation varied from 25 to 82.3%. Moreover, when the authors divided the study subjects into subgroups regarding the ethnicities, they observed a decrease in the heterogeneity of the data ( 7 ). These results illustrate the heterogeneity of the prevalence of BRAF^V600E^ mutation among the different populations, even in the same genetic background, which could explain (at least in part) the conflicting results of the studies regarding the association of BRAF^V600E^ mutation and outcomes.

Our study has several strengths. First, the fact that all patients included in this analysis were treated and followed at the same institution ensures similar therapeutic and follow-up approaches. Moreover, we analyzed the impact of the BRAF^V600E^ mutation not only on oncologic features but also on the clinical outcomes after a median of 8.5 years of follow-up. On the other hand, we are aware that the relatively small sample size could hamper some analysis, especially those regarding the clinical outcomes; therefore, these data should be interpreted with caution. Additionally, our study could not determine a causal relationship between BRAF^V600E^ mutation and outcomes, and BRAF^V600E^ mutation may correlate with some other mutations or confounders, which may turn out to be even more useful prognostic indicators.

In conclusion, we have demonstrated that the current role of the BRAF^V600E^ mutation on the risk stratification of PTC is limited. The inclusion of BRAF^V600E^ mutational analysis in clinical practice increases the costs, whereas the impact of this strategy on patient risk classification or disease outcome prognostication remains uncertain.
